# Huge, invasive, and destructive *Abiotrophia defectiva* endocarditis of the aortic valve and the aortic wall: a case report of an emergency but successful Ross–Konno operation in a child

**DOI:** 10.1093/ehjcr/ytae356

**Published:** 2024-07-23

**Authors:** Vera Cetera, Massimiliano Cantinotti, Elisa Barberi, Vitali Pak

**Affiliations:** Fondazione Toscana Gabriele Monasterio, Heart Hospital, Via Aurelia Sud, Massa 54100, Italy; Fondazione Toscana Gabriele Monasterio, Heart Hospital, Via Aurelia Sud, Massa 54100, Italy; Fondazione Toscana Gabriele Monasterio, Heart Hospital, Via Aurelia Sud, Massa 54100, Italy; Fondazione Toscana Gabriele Monasterio, Heart Hospital, Via Aurelia Sud, Massa 54100, Italy

**Keywords:** *Abiotrophia defectiva* endocarditis, Endocarditis in children, Aortic valve endocarditis, Ross–Konno operation, Case report

## Abstract

**Background:**

*Abiotrophia defectiva* forms Gram-positive cocci, is part of normal oropharyngeal and gastrointestinal flora, and is rarely involved in endocarditis in children population. Its special nutritional requirements and subacute clinical course may delay diagnosis and proper treatment, leading to life-threatening consequences.

**Case summary:**

We report a rare case of huge and destructive *A. defectiva* infective endocarditis (IE) of the aortic valve and the aortic wall in a 3-year-old child, in follow-up after surgical valvuloplasty for congenital aortic stenosis. The child presented at our department with signs of left side hemiplegia. Transthoracic echocardiography showed severe aortic regurgitation due to large vegetation extending to the aortic wall up to the aortic arch. Blood cultures resulted positive for *A. defectiva*. He was initially treated conservatively with antibiotic therapy. Ten days after admission, because of clinical deterioration, he required intubation and an emergency Ross–Konno operation. Despite the critical conditions and highly risky surgery, the child recovered well and was discharged home 5 weeks after the operation.

**Discussion:**

*Abiotrophia defectiva* IE is rare in children. Since 1995, only 16 cases of *A. defectiva* IE have been reported in children, including our case. This pathogen has a higher rate of complications when affecting children rather than adult population. Our case demonstrates that conservative strategy with antibiotics is rarely resolutive in the case of IE caused by *A. defectiva*. Whenever one or more indications for surgery are present, surgical intervention should always be taken into consideration, even if clinical conditions are prohibitive and surgery is at very high risk.

**Learning points**

We report a case of huge and destructive *Abiotrophia defectiva* infective endocarditis in a 3-year-old child and performed a brief review of the literature regarding paediatric population. Based on our experience and considering what is described by other authors, we suggest that the main learning points of this paper are as follows:

- *Abiotrophia defectiva* endocarditis, even if it is a rare pathogen, has high rate of life-threatening complications in children.
- Prompt diagnosis and proper antibiotic treatment are crucial to avoid fatal events.
- Nevertheless, conservative strategy with antibiotics is rarely effective alone. The majority of the cases reported in the literature, including ours, ended up with a surgical solution.
- When one or more indicators for surgery are present, the intervention should not be delayed, even though very risky.
- According to the literature and our experience, surgery is often resolutive, even when performed in a very critical condition.

## Introduction

*Abiotrophia defectiva* is part of the normal flora of the gastrointestinal tract and is rarely involved in infective endocarditis (IE). Due to its atypical clinical presentation and the need for specialized culture media, diagnosis and treatment of IE by this pathogen may be delayed.^1,2^ Compared with other streptococci, IE by *A. defectiva* is known to be more aggressive and predisposed to valve destruction, heart failure, systemic embolization, and death.^2,3^ The majority of patients affected by *A. defectiva* IE require surgical intervention despite treatment with sensitive antibiotics.^2,4^ Our study reports one case of *A. defectiva* IE in a 3-year-old child with a history of aortic valve repair, who had severe neurological complications and successfully underwent Ross–Konno operation.

## Summary figure

**Figure:**
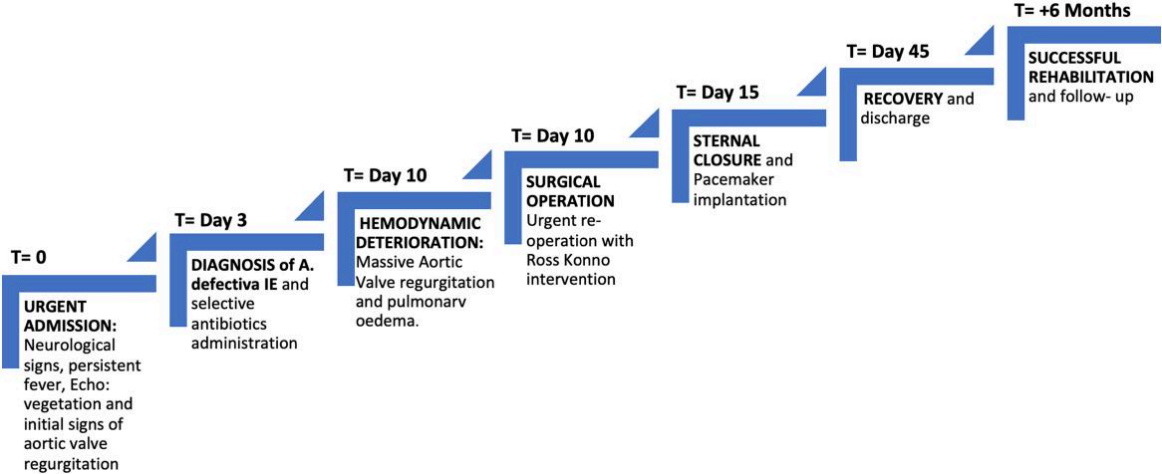


## Case report

**Figure 1:**
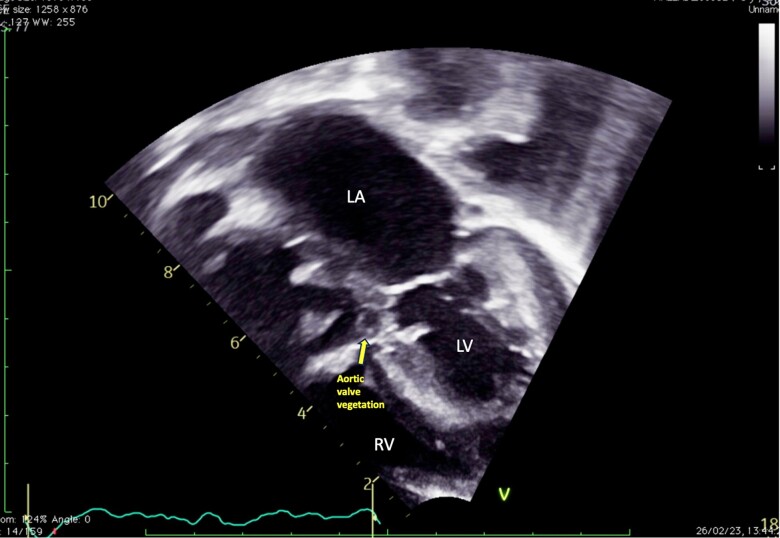
Transthoracic echocardiography performed at admission. Five-chamber view showing aortic valve vegetation.

**Figure 2:**
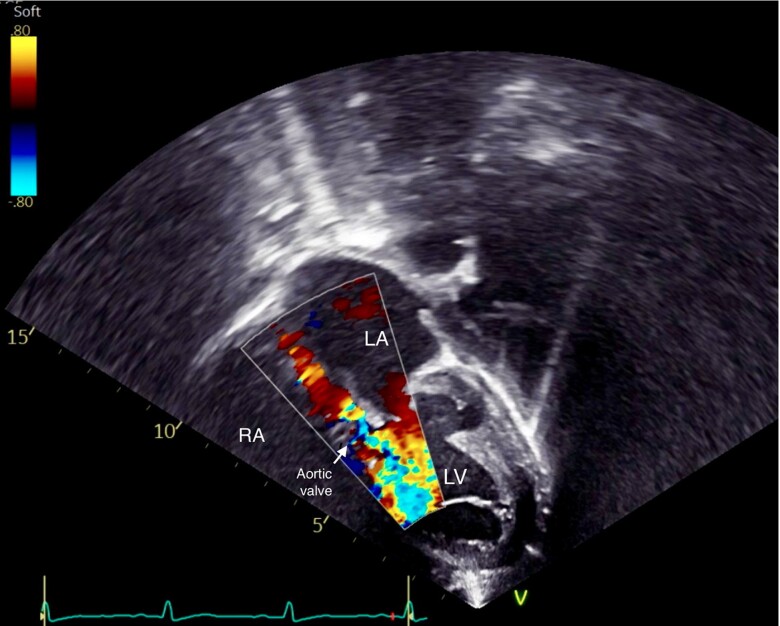
Transthoracic echocardiography images showing massive aortic valve regurgitation on five-chamber views with colour Doppler.

**Figure 3:**
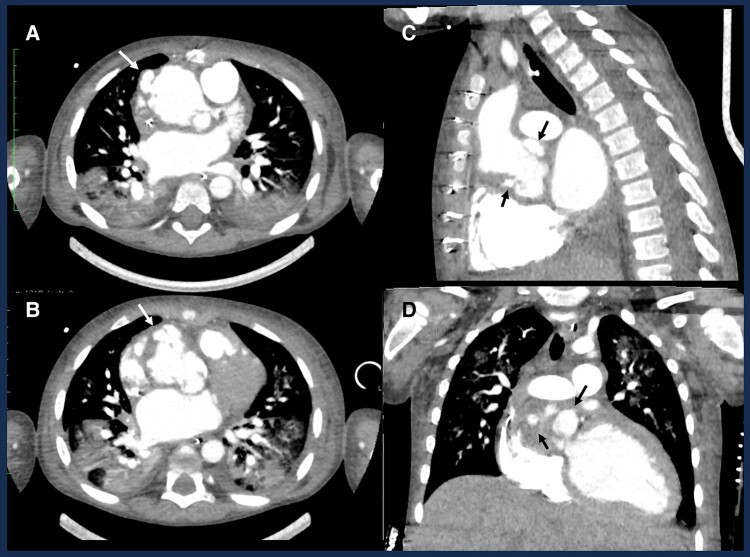
Chest computed tomography scan showing several pseudoaneurysm and abscess (arrows) regarding the aortic root and ascending aorta on axial (*A* and *B*), sagittal (*C*), and coronal (*D*) views.

A 3-year-old male child, in follow-up for congenital aortic stenosis after neonatal surgical valvuloplasty, presented at our department with signs of left side hemiplegia. The parents reported only an isolated episode of toothache a few days prior to the onset of symptoms, which did not require any dental procedure. Upon admission, the child was irritable, his body temperature was 35.6°C, pulse rate of 130 b.p.m., blood pressure of 108/50 mmHg, and oxygen saturation of 98%. Electrocardiography showed moderate sinus tachycardia, normal PR interval and Qtc, left ventricular hypertrophy, and mild intraventricular delay. The transthoracic echocardiography (TTE) revealed moderate aortic regurgitation due to large vegetation on the aortic valve, extending to the aortic root and ascending aorta (see *Figure 1*). The ascending aorta was dilated (diameter: 39 × 38 mm) with multiple pseudoaneurysms on the right anterolateral wall (the largest: 12 × 9 mm of diameter). The size and systolic function of both ventricles were normal. Due to suspicion of cerebral embolization, computed tomography (CT) scan was urgently requested, which revealed a subtotal occlusion of the right median cerebral artery. Laboratory findings showed normal white blood cell count with high levels of C-reactive protein (10 mg/dL). Two separate sets of blood cultures, taken on admission, showed colonies of Gram-positive cocci growing on a blood agar plate. On the third day after admission, *A. defectiva* was identified by matrix-assisted laser desorption/ionization time-of-flight mass spectrometry and the triple antibiotic treatment (vancomycin 100 mg/kg/day + ceftriaxone 80 mg/kg/day + gentamicin 35 mg/day) was started. Although his initial clinical stability led us to a conservative approach, 10 days after the admission, the child manifested a sudden deterioration of clinical conditions and developed signs of heart and lung failure, requiring urgent oral intubation. Transthoracic echocardiography demonstrated massive aortic regurgitation (*Figure 2*) and moderate tricuspid valve regurgitation (TVR) with signs of pulmonary hypertension and pulmonary oedema. Computed tomography chest scan was urgently performed, confirming the TTE findings (*Figure 3*). The heart team thereafter evaluated the critical conditions, young age, and surgical history of the patient and decided to proceed with an emergency Ross–Konno operation. Intraoperative findings confirmed the presence of active endocarditis, probably originating from the aortic annulus and then infiltrating the aortic root and the ascending aorta. Aortic wall appeared to be affected by several pseudoaneurysms bordered solely by adventitial tissue. The pulmonary valve and pulmonary truncus, on the other side, seemed to be free from vegetations or abscess. Several samples of tissue, collected and sent for microbiological analysis, resulted positive for *A. defectiva*. We harvested the pulmonary autograft and sutured it in aortic position and replaced the pulmonary trunk using Contegra® 20 mm conduit. Following the aortic clamp removal, temporary epicardial pacing leads were placed on the epicardium and immediately initiated for complete atrioventricular (AV) block. After a slow weaning and haemofiltration, we were able to close the sternum and move the patient to the post-operative care unit. Overnight, due to progressive signs of right ventricular failure, we reopened the sternum. On the fifth post-operative day, clinical improvements and haemodynamic stability allowed for successful sternal closure. At the same time, considering the persisting complete AV block, we decided to implant a permanent pacemaker. The following post-operative course was uncomplicated. The patient was extubated 2 days after the sternal closure. During his stay, he received 2 weeks of triple antibiotic therapy, then downgraded to vancomycin and ceftriaxone, to complete 5 weeks of intravenous treatment. He showed considerable neuro-motor improvements and was discharged home on 45th post-operative day, with one more month of oral antibiotic therapy (cefixime 100 mg/day). We performed several TTE during his stay, always confirming the good surgical results on the neo-aortic valve and good ventricular function. Residual moderate to severe TVR was also revealed, with no clinical consequences. This pathological finding is the result of TV annulus dilatation and consequent lack of coaptation. At the latest TTE assessment, 6 months after surgery, TVR remained stable, with an estimated systolic pulmonary pressure of 35 mmHg. The child was totally asymptomatic, on medical therapy (diuretic, beta-blocker, and aldosterone antagonist).

## Discussion

*Abiotrophia defectiva* forms Gram-positive cocci, part of normal oropharyngeal flora, that is rarely involved in endocarditis, osteomyelitis, meningitis, brain abscess, and septic arthritis.^1^ According to the literature, *Abiotrophia* spp. are responsible for 4–6% of all cases of streptococcal endocarditis.^2,3^ To the best of our knowledge, 16 cases of *A*. *defectiva* IE have been reported in children population since 1995, including our case (*Table 1*).^4,5–10^ Among these 16 cases, 9 patients (56%) had a history of congenital heart disease (CHD). The patient we reported underwent surgery during the first year of life due to congenital aortic valve stenosis. This may suggest that children with CHD or previous cardiac surgery are more susceptible to contracting *A. defectiva* IE. Notably, in >60% of cases of *A. defectiva* IE, children had dental procedure or oral cavity infection (pharyngitis or suppurative tonsillitis) at the time of diagnosis. In our case, even though the primitive infectious source was not clearly identified, the toothache and the onset of the symptoms, within the following few days, remain extremely suspicious. Strong binding affinity for extracellular matrix proteins and high virulence can explain why *A. defectiva* causes severe complications, such as valve damage or emboli.^11^ Children seem to have a higher rate of complications (69% had embolic events) than adults, where the rate of systemic embolism is 11.8% and mycotic aneurysm is 8.8%.^2^ Our patient unfortunately presented both cerebral embolization and massive valve regurgitation. Considering this important rate of complications, prompt diagnosis and administration of appropriate antibiotics are crucial. Nowadays, the most common antibiotic strategy consists of β-lactam or vancomycin plus gentamicin.^2,12^ However, a recent study demonstrated that *A. defectiva* spp. 95–100% are responsive to third-generation cephalosporins.^13^ At admission, we started a broad-spectrum antibiotic therapy and then adjusted to vancomycin + ceftriaxone + gentamicin. Despite the specific antibiotic treatment, surgery was required in 10 out of the 16 cases reported in the literature. Recurrent embolic events, persistent large vegetation, or severe valvular dysfunction are the principal indications for surgery.^12^ In our experience, the decision to proceed with the surgery was particularly challenging, because of the complexity of the procedure (Ross–Konno operation) and the critical conditions of the child (haemodynamic instability, pulmonary oedema, and recent cerebral embolization). Nevertheless, a radical surgical intervention with delayed sternal closure, in addition to prolonged antibiotic therapy, revealed to be the correct strategy in such a difficult scenario.

**Table 1:** Literature review of *A. defectiva* endocarditis in children

Study	Age	Sex	CHD	Predisposing factor	Embolization	Vegetation	Antibiotics	Surgery
Song *et al.*	15	M	0	0	Cerebral/splenic	MV	AMP + CRO + GEN	No
Song *et al.*	6	M	VSD	0	Septic pneumonia	TV	AMP + CRO + GEN	Yes
Song *et al.*	3.5	F	PA	0	Septic pneumonia	PV	CTX + CRO	No
Song *et al*.	6	F	VSD	0	Septic pneumonia	TV	CRO + GEN	Yes
Chang *et al*.	12	F	0	Dental procedure	Thigh	MV	VAN + GEN → AMP + GEN → CTX + RIF	No
Raf *et al*.	5	M	0	Dental procedure	Foot	MV + AV	VAN + GEN	Yes
Takayama *et al*.	17	M	0	Dental procedure	Septic pneumonia	TV	PEN + GEN	Yes
Bhat *et al*.	11	M	0	n.d.	Cerebral/splenic	MV	AMP/VAN + GEN	Yes
Bhat *et al*.	14	F	0	Dental procedure	0	MV	AMP + GEN	Yes
Torres *et al*.	9	M	VSD	Pharyngitis	0	RV + TV	AMP + GEN	No
Bonura *et al*.	9	F	DX, VSD, AR, LV pseudoaneurysm	Face and oral trauma	Cerebral	Only LV pseudoaneurysm	CRO + GEN	Yes
Du *et al*.	8	F	0	Suppurative tonsillitis	Cerebral	MV + left atrium	VAN + MRP	n.d.
Dong *et al*.	10	F	VSD	Pharyngitis	0	RV + TV	VAN + MRP	Yes
Hayashi *et al*.	9	M	VSD + BAV	Dental procedure	n.d.	n.d.	PEN	No
Krajcar *et al*.	5.5	F	PDA	n.d.	0	0	CRO → cefpodoxime	Yes
Our case	3	M	AS s/p valvuloplasty	n.d.	Cerebral	AV + aortic root + ascending aorta	VAN + GEN + CRO	Yes

VSD, ventricular septal defect; PA, pulmonary atresia; DX, dextrocardia; AR, aortic regurgitation; LV, left ventricle; BAV, bicuspid aortic valve; PDA, patent ductus arteriosus; AS, aortic stenosis; MV, mitral valve; TV, tricuspid valve; PV, pulmonary valve; AV, aortic valve; RV, right ventricle; AMP, ampicillin; CRO, ceftriaxone; GEN, gentamicin; CTX, cefotaxime; VAN, vancomycin; RIF, rifampin; PEN, penicillin; MRP, meropenem; n.d., no data.

## Conclusion

Although *A. defectiva* remains a rare pathogen involved in IE, in the paediatric population, it causes a higher rate of complications. Prompt diagnosis and appropriate antibiotic administration are necessary to avoid life-threatening consequences. However, a conservative strategy is not always resolutive. Based on our experience and considering the good results reported in the literature, we suggest that surgery should not be delayed if one or more indications are present, even in highly risky conditions.

## Data Availability

The data that support the findings of this study are available from the corresponding author, C.V., upon reasonable request.

## Lead author biography

Pediatric cardiac surgeon at Fondazione Toscana Gabriele Monasterio Heart Hospital, Massa, Italy. Graduated from the University of Parma (Italy). Residency in cardiac surgery at the University of Verona (Italy).

## Notes

**Consent:** The authors confirm that written consent for submission and publication of this case report, including the images and associated text, has been obtained from the patient’s next of kin in line with COPE guidance.

**Funding:** None declared.
